# The impact of the Safe Delivery Application on knowledge and skills managing postpartum haemorrhage in a low resource setting: a cluster randomized controlled trial in West Wollega region, Ethiopia

**DOI:** 10.1186/s12978-023-01635-7

**Published:** 2023-06-16

**Authors:** Ann-Marie Hellerung Christiansen, Bjarke Lund Sørensen, Ida Marie Boas, Tariku Bedesa, Wondewossen Fekede, Henriette Svarre Nielsen, Stine Lund

**Affiliations:** 1 Department of Obstetrics and Gynecology, Copenhagen University Hospital Hvidovre, Kettegaard Alle 30, 2650 Hvidovre, Denmark; 2 Department of Clinical Medicine, University of Copenhagen, Blegdamsvej 3B, 2200 Copenhagen N, Denmark; 3 Department of Obstetrics and Gynecology, University Hospital Zealand, Sygehusvej 10, 4000 Roskilde, Denmark; 4 Maternity Foundation, Forbindelsesvej 3, 2100 Copenhagen Ø, Denmark; 5 Global Health Unit, Department of Paediatrics and Adolescent Medicine, The Juliane Marie Centre, Copenhagen University Hospital Rigshospitalet, Blegdamsvej 9, 2100 Copenhagen, Denmark; 6 Department of Neonatology, The Juliane Marie Centre, Copenhagen University Hospital Rigshospitalet, Blegdamsvej 9, 2100 Copenhagen, Denmark

**Keywords:** Mobile health, Maternal health, Postpartum hermorrhage, Ethiopia, Digital health, Childbirth, Midwives

## Abstract

**Background:**

Postpartum haemorrhage is one of the leading causes of maternal mortality in low-income countries. Improving health workers' competencies in obstetric emergencies in low-income settings, has been recognized as an important factor in preventing maternal mortality and morbidity. mHealth interventions in maternal and newborn health care has shown the potential to improve health service delivery. Strong study designs such as randomized controlled trials are missing to estimate the effectiveness of the mHealth interventions.

**Methods:**

Between August 2013 and August 2014, 70 health facilities in West Wollega Region, Ethiopia were included and randomized to intervention or control in a cluster randomized controlled trial. At intervention facilities birth attendants were provided with a smartphone with the SDA installed. Of 176 midwives and “health extension workers,” 130 completed at 12 months follow-up. At baseline and after 6- and 12-months participants were assessed. Knowledge was tested by a Key Feature Questionnaire, skills by an Objective Structured Assessment of Technical Skills in a structured role-play scenario.

**Results:**

Baseline skills scores were low and comparable with a median of 12/100 in the intervention and the control group. After 6 months skills had doubled in the intervention group (adjusted mean difference 29.6; 95% CI 24.2–35.1 compared to 1·8; 95% CI – 2.7 to 6.3 in the control group). At 12 months skills had further improved in the intervention group (adjusted mean difference 13.3; 95% CI 8.3–18.3 compared to 3.1; 95% CI – 1.0 to 7.3 in the control group). Knowledge scores also significantly improved in the intervention group compared to the control (adjusted mean difference after 12 months 8.5; 95% CI 2.0–15.0).

**Conclusion:**

The Safe Delivery App more than doubled clinical skills for managing postpartum haemorrhage among birth attendants making it an attractive tool to reduce maternal mortality.

*Trial registration*: Clinicaltrial.gov Identifier NCT01945931. September 5, 2013.

## Plain Language summary

Maternal mortality caused by postpartum haemorrhage is a major public health concern in many low-income countries. Having access to skilled health care professionals during pregnancy and childbirth can prevent maternal deaths related to postpartum haemorrhage. mHealth interventions like the Safe Delivery App (SDA), a smartphone application, has shown the potential to improve the quality of care in emergency situations related to childbirth in low-income health system settings.

This study examines the SDA as a training/education tool for improving health workers’ competencies and performances in managing postpartum haemorrhage. The SDA contained animated instruction videos on how to prevent and treat postpartum haemorrhage and a list of essential drugs and basic equipment.

In Ethiopia, 70 health facilities and 176 birth attendants were included in a randomized controlled trial. The intervention group received a smartphone with the SDA installed and half a day of introduction to the use of the app. Birth attendants’ skills and knowledge in managing postpartum haemorrhage in the control and the intervention group were tested at baseline, 6 and 12 months after intervention.

Our study found that the SDA is an effective tool to improve and sustain birth attendants’ knowledge and skills in the management of postpartum haemorrhage in a rural, low-resource health system setting in Ethiopia, which confirms findings in other non-randomized studies examining the SDA on the management of postpartum haemorrhage.

## Background

Globally an estimated 295,000 women die each year due to pregnancy and childbirth almost exclusively in resource constrained settings [1]. The main cause of maternal mortality is postpartum haemorrhage (PPH) affecting as many as one in three women giving birth in low-income countries [2]. There are three main causes of PPH; uterine atony, retained products of pregnancy and trauma to the birth canal. Uterine atony is the most common cause of PPH [3]. The safe management of PPH is well known and like all emergencies the success depends on prompt response by a well-coordinated team. Basic resuscitative measures must be taken to secure airway, breathing and circulation, mechanical manoeuvres like bimanual uterine compression and aorta compression as well as a number of oxytocic drugs can control bleeding caused by uterine atony, and a number of minor surgical interventions like manual removal of retained products and suturing of lacerations must be mastered [3]. The United Nations have stated seven signal functions of Basic Emergency Obstetric and Neonatal Care (BEmONC) that must be provided by any skilled birth attendant (SBA). The signal functions are provision of parental antibiotics, provision of uterotonic drugs, administration of parental anticonvulsants, manual removal of the placenta, removal of retained products, assisted vaginal delivery and neonatal resuscitation [4]. It has been demonstrated how the response to obstetric emergencies within health facilities in low-income countries is often severely deficient endangering the health of women and their newborn, in reality the so-called ‘skilled birth attendants’ are often not able to provide ‘skilled birth attendance’ [5–8]. However, quality assurance of emergency obstetric care in low-income countries is difficult to extend to the peripheral parts of the health system where the majority of births take place, and obstetric emergencies are to be managed by health professionals working in rural areas supported by poor infrastructure [9]. Promotion of health services via mobile health (mHealth) like mobile phones has been suggested as a mean to bridge this outreach gap [10–12]. The Safe Delivery App (SDA) is an emergency obstetric care training aid for skilled birth attendants working isolated in peripheral health facilities in low-income countries. The objective of this study was to assess the impact of the SDA on birth attendants’ knowledge and skills managing postpartum haemorrhage.

## Methods

### Study design

The study was a pragmatic cluster randomized controlled trial, carried out between August 2013 and August 2014 at 70 health facilities in five districts in Oromiya Region, West Wollega Zone in Ethiopia. At the time of the trial the population was approximately 1,600,000 of which 90% lived in rural areas engaged in subsistence agriculture. The zone had 21 districts, of which 19 were rural. The zone had five hospitals (three governmental and two non-governmental), 53 health centres and 440 health posts (governmental). The area was characterized by long distances between villages and nearest towns and health facilities and a lack of infrastructure. The study was performed in the five districts of Nole Kaba, Haru, Homa, Genji and Gimbie. Utilization of maternal health services was generally low. In 2012 there were 2231 deliveries at the two hospitals, 1031 deliveries at health centres and 512 deliveries at health posts. The remaining births took place in villages unsupervised by trained health personnel. Only 30% receive antenatal care from skilled provider and less than 10% deliver supervised by a skilled birth attendant [13]. The health facility was the unit of randomization for either the intervention or control with approval from the Ethiopian Ministry of Health (with ethical clearance obtained from the Oromiya Regional Health Bureau May 7th 2013). clinicaltrials.gov Identifier NCT01945931.

### Participants

All hospitals, health centres and health posts in the five districts were selected for the study and assigned by simple random allocation to either the smartphone intervention or the control group. Health facilities with five or more deliveries in 2012 were eligible for inclusion. The study population were the midwives and health extension workers (HEWs) attending deliveries at health facilities eligible for randomization and who provided individual informed consent for participation. Health staff who were retired or were deployed to another health facility during the study period were excluded. One health facility was excluded as it had neither a midwife nor a HEW employed. In Ethiopia ‘health extension workers’ are the frontline workers, usually working in the most remote and rural areas. Their level of education is minimum one year in general health care including basic maternal health. They are not categorized as “skilled birth attendants”, though they are often the only persons available to manage complications in the remote places they work. All participants were assured confidentiality and that any participation was voluntary. Person-related information like name and place of employment which could violate anonymity of the participants, was kept strictly apart from the data collection forms under double lock.

### Randomization

Health facilities were stratified by level of care and district and allocated to either intervention or control using a computer-generated list of random numbers. Due to the nature of the intervention, masking of participants and research assistant was not possible. Using the health facility rather than individual health providers as the unit of randomization was decided to avoid contamination, as it would be difficult to prevent staff at the same facility to share the SDA.

### Intervention

**Fig. 1:**
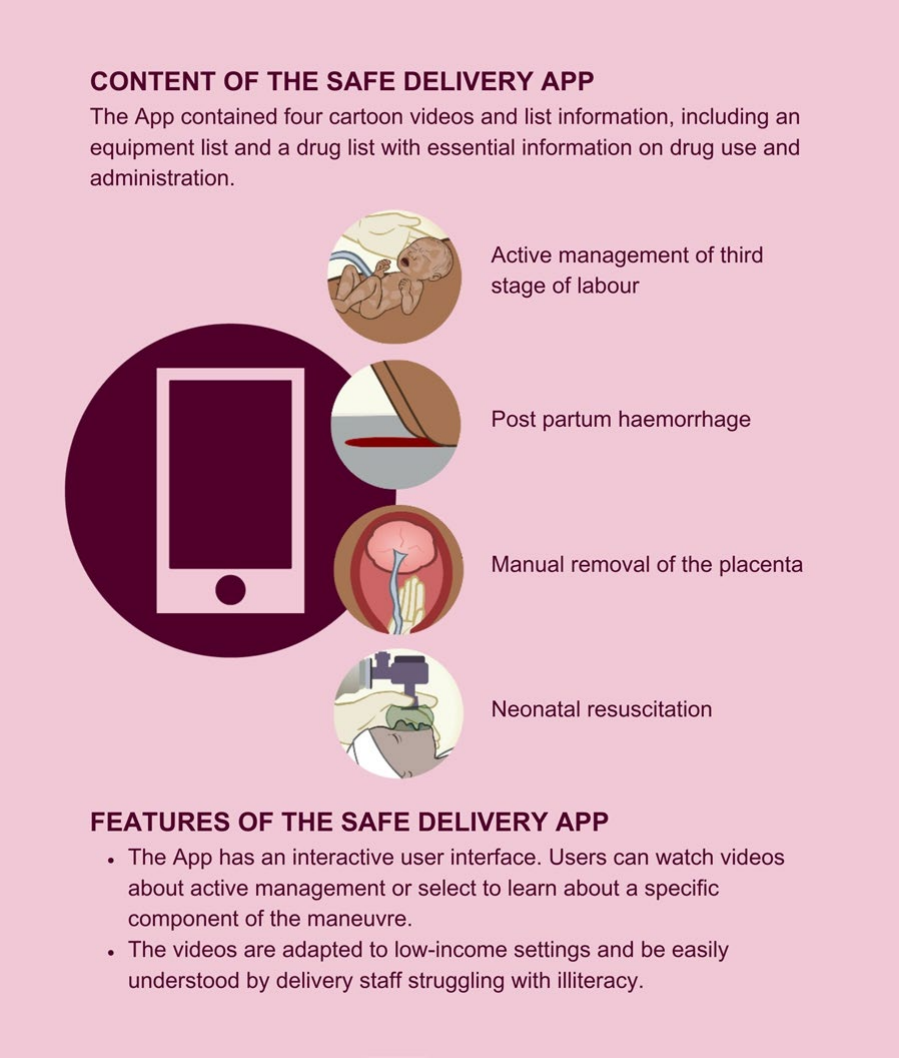
The Safe Delivery App. Printed with permission from Maternity Foundation

The SDA is a BEmONC training aid developed by two researchers from University of Southern Denmark and University of Copenhagen (second and last author), the non-governmental organization Maternity Foundation and Visikon, a private company specialized in visual health information. The app contained four cartoon videos lasting four to eight minutes about active management of the third stage of labour to prevent PPH, the management of manifest PPH, the removal of a retained placenta and neonatal resuscitation (Fig. 1).

The films had a dual language function, either English (the working language at the health facilities) or the local language Oromiffa. It also had a list of essential drugs and a list of basic equipment. Every day the user received notifications to encourage the use of the app. The app had a tracking function where the use of the different content was registered with GPS coordinates. Smart phones with the SDA preloaded including charger and earphones were provided for the health providers in the intervention group. A half-day introduction to the use of the phone and the app was given. This however did not include clinical skills training of any kind. Participants were strictly asked not to share or show the app to other health providers from other health facilities. Beforehand health facilities were assured to have basic equipment and drugs.

After this trial a full version of the app has been developed including all seven BEmONC signal functions which can be downloaded for free at (https://itunes.apple.com/us/app/safe-delivery/id985603707?mt=8 (i-phone) or https://play.google.com/store/apps/details?id=dk.maternity.safedelivery) (android).

Participants’ skills and knowledge were assessed at baseline and at 6- and 12-months follow-up. The skills and knowledge tests were performed by two research assistants, both experienced Ethiopian midwives, who were trained in performing test-scores in structured role-play scenarios with the aid of a pelvic, uterine and neonatal mannequin and a selection of relevant equipment and drugs. One directed the scenario while the other did the observation and scoring. The PPH scenario lasted 10–15 min and was the same for all tested persons. After the scenario, the two research assistants would go through the scoring and agree for each item. The knowledge test was performed on the same day as the skills test. It took place in a classroom setting with no access to any aid and took approximately 1 h. There was an option to have it in English or Oromiffa.

### Outcomes

**Fig. 2:**
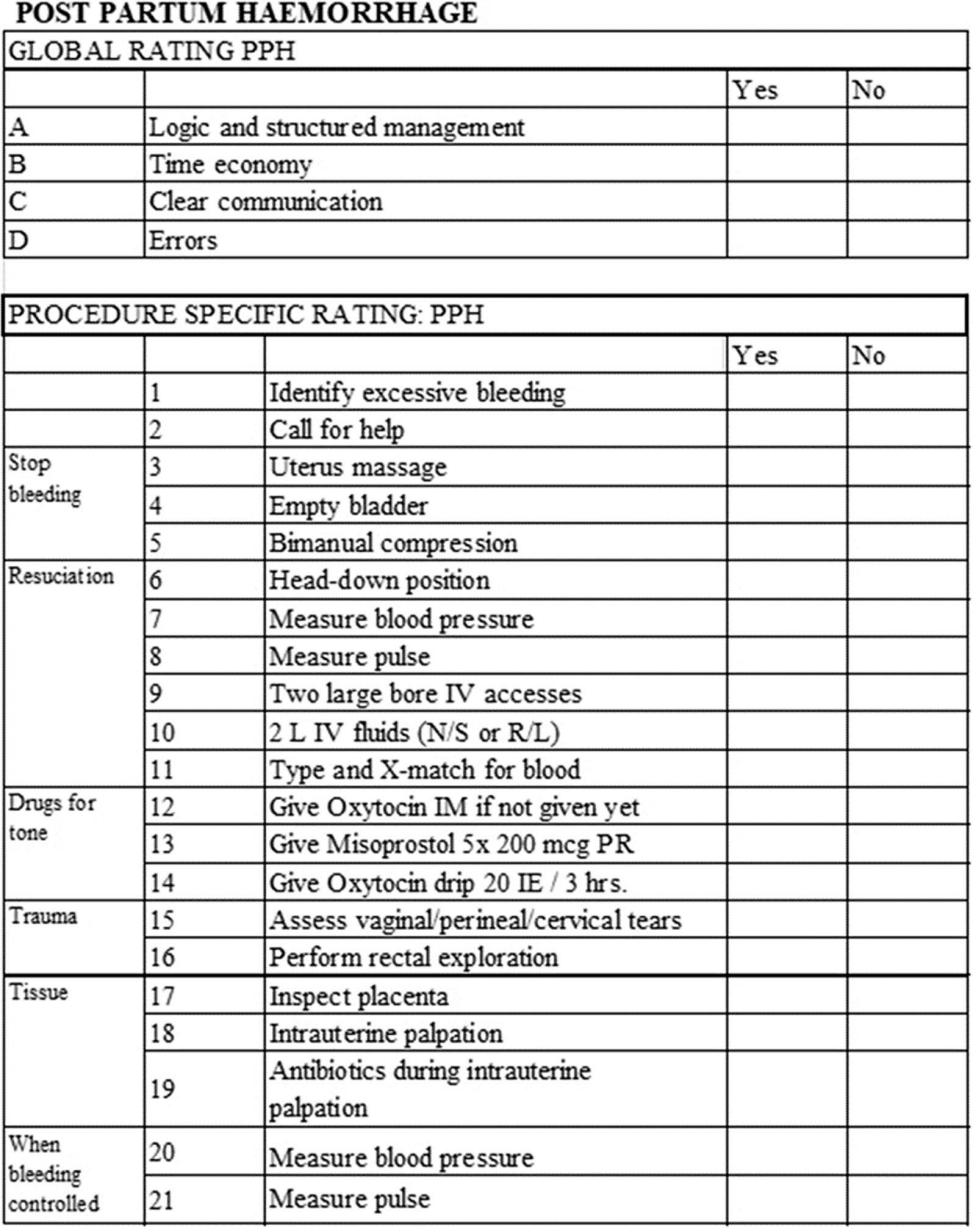
Skills test (objective structured observation of technical skills) observation form

Outcomes were total scores in skills and knowledge tests for management of PPH. As no validated tests useful for the purpose was identified at the planning of the study, two new tests were developed by the clinicians and researchers in the SDA group based on existing guidelines and standards of care in management of PPH. The content validity was assured through a structured review by an expert group whereby three levels of competency from beginner to expert were assessed using the developed tool with significant ability to differentiate. To assess skills, role-play scenarios were conducted with each included health worker individually, and an ‘Objective Structured Assessment of Technical Skills’ (OSATS) scale was developed including all the identified key points and “global scores” related to the overall impression of the performance. Full score was given if a key point was performed or mentioned spontaneously and correct, ½ a point was given if prompting was needed or if only partly correct, otherwise no points was given (Fig. 2).

**Fig. 3:**
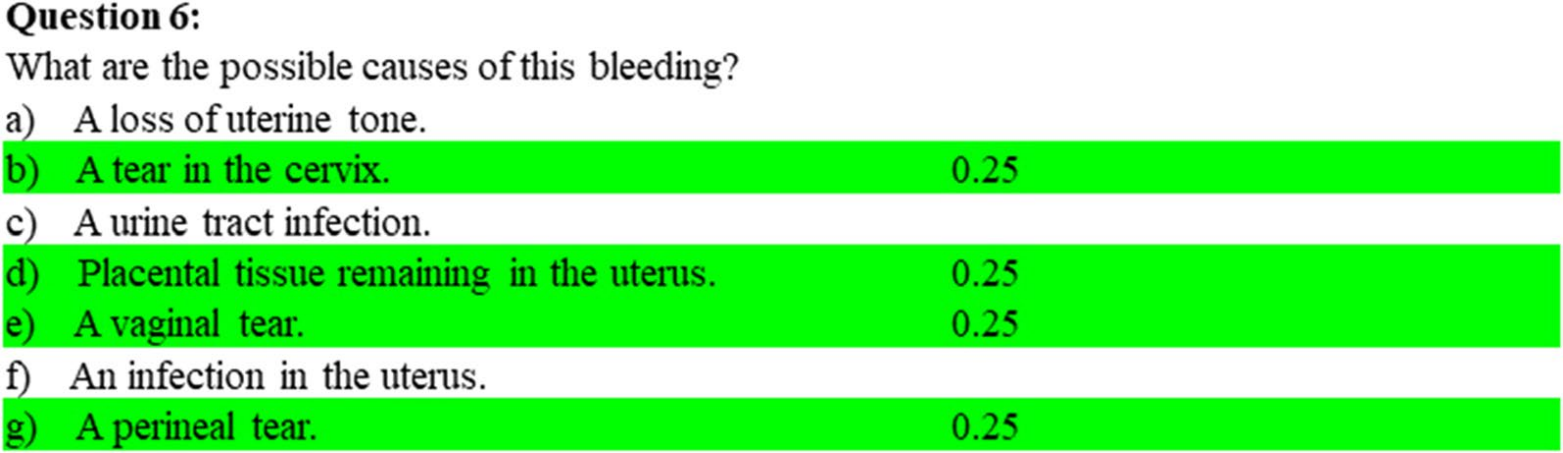
Knowledge test (key feature questionnaire) example of question based on a case. Green marked answer possibilities are correct answers

To test knowledge a Key Feature Questionnaire (KFQ) was prepared with case-based questions related to clinical situations covering all important key points. The number of answer possibilities varied, one or more would be correct and score positive and some would be wrong and would, if constituted malpractice, deduct all marks related to that question (Fig. 3).

Direct measuring of PPH at deliveries at the health facilities during the study period was considered and reported as the primary outcome for clinicaltrials.org. However, it proved unmanageable to oversee a reliable data collection at so many health facilities often situated in remote parts of the study area. The quality of the data was found to be very low and therefore further efforts to pursue this outcome was abandoned.

### Statistical analysis

With a power of 80 and 95% confidence intervals and an estimated baseline score of 40% and an increase to 60% in the intervention group, a sample size of 114 was needed.

Scores for the OSATS and KFQ were double entered in SPSS (version 23, SPSS Inc.) from paper forms by an independent team.

A linear mixed effect model was used to compare the differences in skills and knowledge in intervention and control groups. The model accounts for the correlation between health care workers from the same facility by including health care facility as a random factor. The intervention status was included as a fixed effect. Adjustments were made for individual factors strongly correlating with the outcome; experience with smartphones, profession and number of deliveries. Baseline data was used as covariate (linear effect) and the scores after 6 and 12 months were used as the dependent variables in two separate analyses. The estimated intervention effect is reported as the difference between the intervention and the control group in scores from baseline to 6 and 12 months and between 6 and 12 months. The differences are presented as mean difference in score with a 95% confidence interval as percentages of maximum score achieved, hence both knowledge and skills tests have a range of 0–100%. A significant difference in mean score was considered if the 95% confidence interval was above zero. We used the CONSORT checklist when writing our report [14].

## Results

**Fig. 4:**
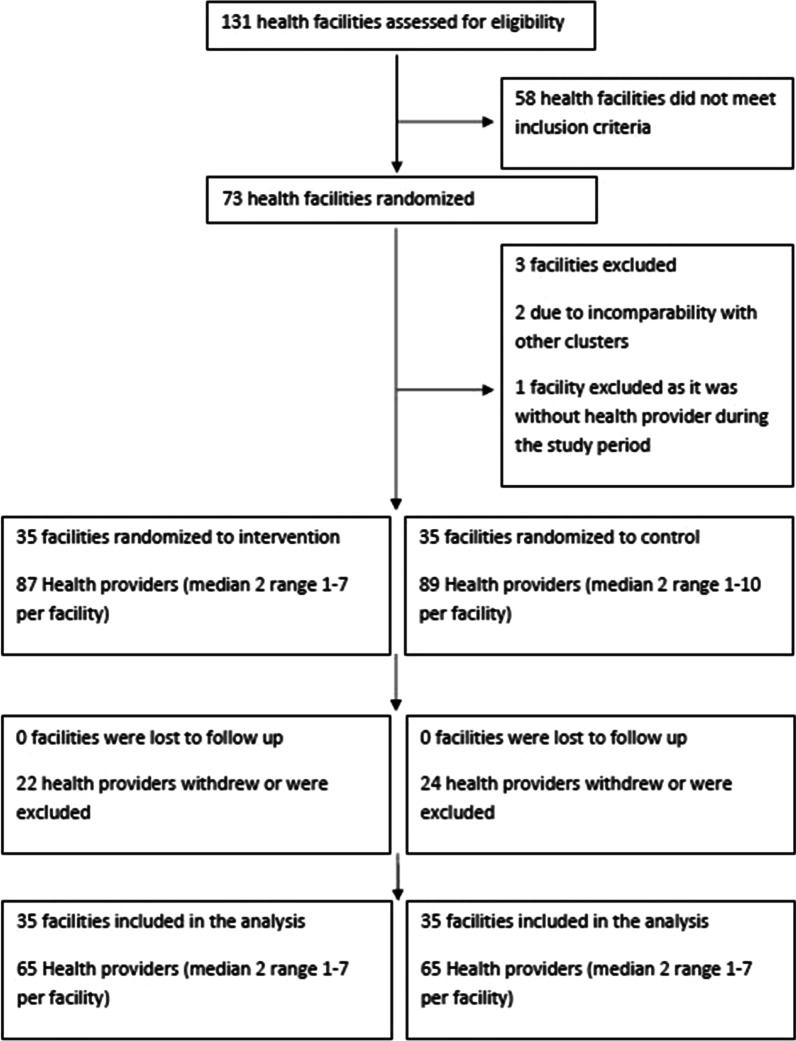
Flow chart

Between August 22, 2013 and August 30, 2014, a total of 130 (74%) of 176 midwives and health extension workers at two hospitals, 13 health centers and 58 health posts were included in the study. Of these, 46 dropped out during the study, mainly because of transfers to other duty stations (n = 24). Two went for educational upgrade, while one was jailed accused of malpractice. A total of 130 (74%) health workers (32 midwives, 88 health extension workers, 8 unknown) completed the 6- and 12 months follow up, 65 in each arm, and are included in the following analysis (Fig. 4).

Background parameters for the participants (Table 1) showed no significant differences between control and intervention groups.

**Table 1:** Background parameters participants

	Intervention	Control
	(n = 65)	(n = 65)
Educational level		
Health extension worker	47 (74.6%)	41 (69.5%)
Clinical nurse or midwife	16 (25.4%)	18 (30.5%)
Number of deliveries past month		
1–5	47 (83.9%)	38 (64.4%)
> 5	9 (16.1%)	21 (35.6%)
Knowledge of smartphone		
Tried using one	15 (23.1%)	26 (40.0%)
Never tried using one	50 (76.0%)	39 (60.0%)
Workplace		
Hospital	5	7
Health Centre	13	12
Health Post	45	39
Missing data	2	7
Gender		
Male	8	4
Female	55	55
Missing data	2	6
Age		
< 25	54	46
≥ 25	9	13
Missing data	2	6

The baseline scores for skills were not normally distributed, with a median of 12/100 in the intervention group (range 0–72, SD 18.2) and 12/100 for the control group (range 0–66, SD 21.1). The increase in skills scores from baseline to 6 and 12 months and from 6 to 12 months were normally distributed with significant improvements for all three time periods. After 6 months, the increase in mean score was 29.6/100 with a further significant increase during the following 6 months of 13.3/100. The control group did not significantly increase skills for the first 6 months while there was a significant improvement of 4.9/100 from baseline to 12 months. The difference in increase in scores between the intervention and the control group was significant for all the time periods assessed, indicating an effect of the SDA of a more than doubling of the skills scores from baseline (Table 2).

**Table 2:** Skills and knowledge scores at 0, 6, and 12 months

	Intervention (95% c.i.)	Control (95% c.i.)	Difference between intervention and control (95% c.i.)
Skills			
0 versus 6 months			
Unadjusted	29.9 (24.7 to 35.2)	1.6 (− 2.8 to 6.0)	28.3 (21.5 to 35.2)
Adjusted	29.6 (24.2 to 35.1)	1.8 (− 2.7 to 6.3)	27.8 (20.5 to 35.0)
6 versus 12 months			
Unadjusted	12.6 (7.7 to 17.4)	3.6 (0.4 to 7.7)	8.9 (2∙6 to 15∙3)
Adjusted	13.3 (8.3 to 18.3)	3.1 (− 1.0 to 7.3)	10.2 (3∙4 to 16∙9)
0 versus 12 months			
Unadjusted	42.5 (37.1 to 47.9)	5.2 (0.7 to 9.7)	37.3 (30.3 to 44.3)
Adjusted	42.9 (37.2 to 48.6)	4.9 (0.2 to 9.7)	38.0 (30.4 to 45.6)
Knowledge			
0 versus 6 months			
Unadjusted	8.9 (4.3 to 13.5)	1.7 (− 2.2 to 5.5)	7.3 (1.3 to 13.3)
Adjusted	8.5 (3.8 to 13.3)	1.9 (− 2.0 to 5.9)	6.6 (0.3 to 13.0)
6 versus 12 months			
Unadjusted	4.2 (− 0.1 to 9.4)	0.3 (− 3.3 to 3.8)	3.9 (− 1.6 to 9.4)
Adjusted	3.0 (− 1.2 to 7.2)	1.1 (− 2.4 to 4.6)	1.9 (− 3.4 to 7.5)
0 versus 12 months			
Unadjusted	13.1 (8.3 to 17.9)	1.9 (− 2.1 to 5.7)	11.2 (4.9 to 17.4)
Adjusted	11.5 (7.7 to 16.4)	3.0 (− 1.0 to 7.1)	8.5 (2.0 to 15.0)

Maximum score = 100%

Adjusted for within-cluster effect and significant variables associated with outcome

Knowledge scores were normally distributed with an adjusted mean score at 12 month follow-up of 39.1/100 (95% c.i. 34.8–43.4) in the intervention group and 32.1/100 (95% c.i. 28.5–35.7) in the control group. This difference was significant with a mean difference of 7.0 (95% c.i. 1.3–12.8). In the intervention group there was a significant improvement in knowledge scores from baseline to 6 months that was sustained after 12 months, while there was no significant change from 6 to 12 months. There was no significant change for any of the time periods for the control group. The difference in change between the groups was significant (Table 2).

## Discussion

In this randomized controlled trial, including 130 mid-and low-level health providers in a rural setting in Western Ethiopia, we have demonstrated that the SDA, an mHealth intervention, was associated with an increase in knowledge and particularly skills managing PPH. Notably we also found an increase in the learning curve up to 12 months from being introduced to the app. These findings are important as they address a main cause of maternal mortality and morbidity and indicate that an mHealth tool might be an important steppingstone for bridging the outreach gap in improving the quality of emergency care in especially the peripheral rural parts of the health system.

The use of mHealth in low-and middle-income countries on maternal and neonatal care has been tested in a growing number of studies, however studies are often of poor methodological quality and few studies deal with mHealth as education and training of health professionals especially within emergency obstetric care [11, 15, 16]. An observational study from Uganda improved adherence to good clinical practice in relation to neonatal resuscitation from 46% to 94% and a self-reported high perceived improvement in quality of newborn care, was found in a study from Malawi [17, 18]**.**

Improved competencies in health workers’ skills and knowledge on PPH management, have been examined in other SDA trials from Rwanda and Congo, and our findings are consistent with the documented effect of the SDA intervention in these studies [19–21]. The pre-post intervention study from Rwanda found a significant increase in knowledge and skills scores on PPH management in the SDA intervention group 6 months after the intervention [19], and the pilot cluster RCT from Congo found a significant increase in PPH knowledge scores after three months of intervention in the SDA group [21]. A second study from Rwanda found a significant reduction in unstable maternal outcomes (persistent bleeding requiring referral to hospital) following PPH management in the SDA intervention group [20]. Thomsen and colleagues reported that Ethiopian health workers experienced and perceived the SDA as a useful tool to improve or retain skills and knowledge and to manage obstetric complications with more confidence [22].

A RCT study from Ethiopia also investigated the effect of the SDA and found a non-significant reduction in perinatal mortality and an increase in skills and knowledge in skilled birth attendants for neonatal resuscitation at 6 and 12 months after the intervention [23]. The study from Ethiopia and Rwanda are one of very few trials examining the direct effect of an mHealth obstetric emergency intervention on clinical outcome. In general, the direct impact of mHealth training interventions on maternal and neonatal outcomes are rarely examined [15, 16].

Conventional BEmONC training courses vary in length from a few days to several years [24]. In sub-Saharan Africa a three weeks course developed by JHPIEGO is widely promoted [25]. The documentation for the quality of these trainings is of varying quality. They are often assessed by “reaction,” “knowledge” and/or “skills” and the impact on clinical outcome is rarely assessed [24, 26]**.** An intervention study assessing a short course BEmONC training at a referral hospital in Tanzania demonstrated a significant reduction in the incidence of PPH from 32.9% to 18.2%, and severe PPH from 9.2% to 4.3% [27].

A cluster RCT from Nigeria examined the effect of simulation-based, low-dose, high frequency training plus mobile mentoring (LDHF/m-mentoring) compared to traditional group-based training (TRAD) on health workers’ knowledge and skills competencies on BEmONC with mixed results [28]. They found that the TRAD intervention had significantly higher knowledge scores at baseline and at immediate post-training assessment compared to the LDHF/m-mentoring intervention. Moreover, they found that that the overall mean score for knowledge at 3 months post training was equal for both training groups and that the overall skills scores had declined since immediate post-training assessment. At 12 months, knowledge scores were equal in both arms, but skills scores were significantly higher in the LDHF/m-mentoring arm [28].

Tools to measure training impact are often not validated or standardized, rather are they constructed for the occasion. Different training modalities were compared to each other in few studies we could identify [28–30], and evidence has shown that short competency-based trainings in BEmONC can result in significant improvements in knowledge and skills [24]. Likewise, it is difficult to compare the demonstrated effect of the SDA compared to conventional trainings. However, the almost three-times increase in skills has not been reported for any other BEmONC training. For example, improved skills for managing PPH was 49–70% among 5939 birth attendants in sub-Saharan Africa after a three days BEmONC training [31]. Retention after training is often not assessed in low-resource settings [24], though the impact is considered to fade with time so that refresher training is recommended at regular intervals [32].

It is remarkable that the small-dose, high-frequency training by the SDA demonstrates full retention of skills and knowledge, and even an increasing instead of declining learning curve up to 12 months after its introduction.

Most studies about the quality of BEmONC are observational and concern referral rather than outreach health facilities [33]. A cluster randomized controlled trial (RCT) at referral hospitals in Senegal and Mali demonstrated a reduced maternal mortality after a mixed intervention including maternal death reviews and training in BEmONC [34].

Financial and qualified workforce restraints are pointed out as main barriers to provision of safe delivery care [35]. The here presented findings suggest that the SDA and other validated mHealth tools might be very effective to address these challenges in low resource health systems. mHealth seem to have an important role to play in efforts to reduce maternal and perinatal mortality and morbidity. However, the success probably depends on contextual factors such as strategies for dissemination and support by national health authorities [36]. In 2021 it was estimated that there were 350,000 health related apps, varying in content and quality [37]. For strategies for disseminating and sustaining the SDA qualitative observational research is needed to identify barriers and facilitating factors to its use from organizational and clinical work perspectives as well as cost-effectiveness.

Weaknesses inherent to the study design can be discussed. The randomization at health facility level was chosen to avoid that health providers in the control group had access to the SDA. We had no observations that this took place. Randomization was made by district with the aim to make intervention and control facilities as comparable as possible regarding infrastructure. Subsequent statistical control of within-cluster correlation and confounders was performed. Blinding of participants was impossible due to the nature of the intervention, knowledge tests were blinded as the scoring was done without knowledge of the health facility, though skills testing was unblinded. This may have caused information bias. Test enhanced learning could explain part of the results though the control group showed only slight and generally non-significant increase in skills- and knowledge score. Extrapolation of these findings to other settings should be cautious as organization of the health system and quality assurance might vary much between different countries, even between different health facilities. Furthermore, the increase in skills and knowledge in test situations might not cause improved management and clinical outcomes in real work situations as this may depend on other factors such as an “enabling environment” hereunder availability of drugs, equipment, staffing and supervision. All this said, it remains that this is one of few randomized controlled trials in a low-income setting to demonstrate a clear effect of an mHealth intervention within a health topic of major importance.

## Conclusion

The SDA was an effective mean to support and sustain health workers’ knowledge and skills in management of postpartum haemorrhage up to 12 months after introduction. The findings are highly relevant for health workers working in resource constraints settings, where quality of care is challenged by lack of continuing educational programs. Further research is needed in order to assess the direct effect of mHealth training tools on large-scale implementation and clinical outcomes.

### The SDA today

Based on the study and a multitude of other research studies documenting its impact, the Safe Delivery App attracted interest from a range of partners and was scaled up globally.

Today, the App is the main component in Maternity Foundations Safe Delivery + programme, which also consists of online and on-the-ground trainings of healthcare workers. In total, more than 300,000 healthcare workers have been reached across more than 40 countries, and the App is available in more than 30 language versions. Through a partner-driven model of implementation, the App is integrated into national education and training programmes, pre-service curricula, and with other digital solutions. This ensures sustainability and further enhances scalability of the App in a cost-effective way.

## Data Availability

The datasets used and/or analyzed during the current study are available from the corresponding author on reasonable request.

## Abbreviations

BEmONC: Basic Emergency Obstetric and Neonatal Care
KFQ: Key Feature Questionnaire
LDHF/m-mentoring: Low-dose, high frequency training plus mobile mentoring
mHealth: Mobile health
OSATS: Objective Structured Assessment of Technical Skills
PPH: Postpartum haemorrhage
SBA: Skilled birth attendant
SDA: Safe Delivery App
TRAD: Traditional group-based training
