# Correction: The impact of the Safe Delivery Application on knowledge and skills managing postpartum haemorrhage in a low resource setting: a cluster randomized controlled trial in West Wollega region, Ethiopia

**DOI:** 10.1186/s12978-023-01646-4

**Published:** 2023-08-16

**Authors:** Ann-Marie Hellerung Christiansen, Bjarke Lund Sørensen, Ida Marie Boas, Tariku Bedesa, Wondewossen Fekede, Henriette Svarre Nielsen, Stine Lund

**Affiliations:** 1 Department of Obstetrics and Gynecology, Copenhagen University Hospital Hvidovre, Kettegaard Alle 30, 2650 Hvidovre, Denmark; 2 Department of Clinical Medicine, University of Copenhagen, Blegdamsvej 3B, 2200 Copenhagen N, Denmark; 3 Department of Obstetrics and Gynecology, University Hospital Zealand, Sygehusvej 10, 4000 Roskilde, Denmark; 4 Maternity Foundation, Forbindelsesvej 3, 2100 Copenhagen Ø, Denmark; 5 Global Health Unit, Department of Paediatrics and Adolescent Medicine, The Juliane Marie Centre, Copenhagen University Hospital Rigshospitalet, Blegdamsvej 9, 2100 Copenhagen, Denmark; 6 Department of Neonatology, The Juliane Marie Centre, Copenhagen University Hospital Rigshospitalet, Blegdamsvej 9, 2100 Copenhagen, Denmark

**Correction: Reproductive Health (2023) 20:91** **https://doi.org/10.1186/s12978-023-01635-7**

After publication of this article [1], the authors reported that in the ‘Methods’-section, subsection ‘Intervention’, first sentence, it should say ‘second and last author’ instead of ‘first and last author’.

The original article [1] has been corrected.
